# Distribution Drivers of the Alien Butterfly Geranium Bronze (*Cacyreus marshalli*) in an Alpine Protected Area and Indications for an Effective Management

**DOI:** 10.3390/biology11040563

**Published:** 2022-04-07

**Authors:** Emanuel Rocchia, Massimiliano Luppi, Federica Paradiso, Silvia Ghidotti, Francesca Martelli, Cristiana Cerrato, Ramona Viterbi, Simona Bonelli

**Affiliations:** 1 Alpine Wildlife Research Centre, Gran Paradiso National Park, Via Pio VII 9, 10135 Turin, Italy; ema.rocchia@gmail.com (E.R.); silvia.ghido@gmail.com (S.G.); cri.entessa@virgilio.it (C.C.); ramona.viterbi@pngp.it (R.V.); 2 Department of Life Sciences and Systems Biology, University of Turin, 10123 Turin, Italy; massimiliano.luppi85@gmail.com (M.L.); simona.bonelli@unito.it (S.B.); 3 Department of Geography and Environmental Science, Northumbria University, Ellison Pl, Newcastle upon Tyne NE1 8ST, UK; francesca.martelli@northumbria.ac.uk

**Keywords:** allochthonous insect, species distribution, *Pelargonium*, N-mixture models, MaxEnt, mitigation strategy, citizen science, climate change

## Abstract

**Simple Summary:**

*Cacyreus marshalli* is strictly dependent on its host plant (*Pelargonium* spp.), which is widely cultivated as an ornamental plant in mountain areas. An experiment demonstrated that the butterfly is able to develop on some wild geraniums, too, making mountain areas highly at risk for a potential expansion to natural habitats. We therefore decided to carry out research in a protected mountain area (Gran Paradiso National Park), focusing on the drivers which determine the distribution of *C. marshalli* using data provided by either an opportunistic approach or a rigorous survey protocol. The data collected via the planned survey were more informative than the opportunistic observations, which were few and narrow. We suggest investing more in citizen science projects and combining them with a designed protocol according to an integrated approach. We observed that *C. marshalli* distribution is strictly linked to host plant availability but is constrained by cold temperatures, although *Pelargonium* spp. are abundant. The temperature increase scenario showed an increase of butterfly abundance, but halving of the host plant population could drive the rate of infestation to return to what it was previously, excluding a countertrend in some high-altitude sites. It is therefore important to test management actions designed to control alien species before implementing them.

**Abstract:**

*Cacyreus marshalli* is the only alien butterfly in Europe. It has recently spread in the Gran Paradiso National Park (GPNP), where it could potentially compete with native geranium-consuming butterflies. Our study aimed to (1) assess the main drivers of its distribution, (2) evaluate the potential species distribution in GPNP and (3) predict different scenarios to understand the impact of climate warming and the effect of possible mitigations. Considering different sampling designs (opportunistic and standardised) and different statistical approaches (MaxEnt and N-mixture models), we built up models predicting habitat suitability and egg abundance for the alien species, testing covariates as bioclimatic variables, food plant (*Pelargonium* spp.) distribution and land cover. A standardised approach resulted in more informative data collection due to the survey design adopted. Opportunistic data could be potentially informative but a major investment in citizen science projects would be needed. Both approaches showed that *C. marshalli* is associated with its host plant distribution and therefore confined in urban areas. Its expansion is controlled by cold temperatures which, even if the host plant is abundant, constrain the number of eggs. Rising temperatures could lead to an increase in the number of eggs laid, but the halving of *Pelargonium* spp. populations would mostly mitigate the trend, with a slight countertrend at high elevations.

## 1. Introduction

The introduction of alien species is one of the most important causes of biodiversity loss. Their impact on native ecosystems is even more problematic in protected areas, where it becomes crucial to understand the distribution of invasive species to undertake management strategies that can limit the spread and potential risks to biodiversity [1,2,3]. 

Originally from a wide area of southern Africa (Zambia, Mozambique, Zimbabwe, Botswana, South Africa, Swaziland [4,5]), *Cacyreus marshalli* (Butler, 1898) (Lepidoptera: Lycaenidae) was first introduced in Europe through the trade of *Pelargonium* (Geraniaceae), and it currently represents the only non-native butterfly species among 482 European species [6]. In Europe, despite the abundance of *Geranium* and *Erodium* species (spontaneous Geraniaceae), only the genus *Pelargonium* (not native to Europe) serves as a host plant, facilitating the spread of the species through the commerce of ornamental plants [7]. No recording of eggs laid on wild European *Geranium* spp. has been reported. However, a previous research demonstrated the ability of *C. marshalli* to develop on some native plant species in controlled conditions [8] and to overcome natural barriers such as trees to find host plants [9]. Consequently, the risk of naturalisation of this species is high, posing an important threat for autochthonous *Geranium*-consuming butterflies (i.e., *Eumedonia eumedon* and *Aricia* spp. [8]). In Italy, and particularly in the Alpine area, *Pelargonium* cultivars are widely used as ornamental plants in both private and public areas because of their colourful flowers and resistance to drought and cold weather. Thus, the Alpine areas are zones with a potential elevated presence of *C. marshalli* with a high risk of naturalisation. For these reasons, the Gran Paradiso National Park (GPNP—where *C. marshalli* was detected for the first time in 2015) activated a monitoring program to assess the distribution of this species, involving standardised monitoring projects and opportunistic data collection through citizen science (CS).

GPNP is located in the Western Alps, an area for which models predict significant climate warming in the next decades [10], which would have a complex impact on Alpine ecosystems [11,12]. Heat-sensitive species usually redefine their range, shifting towards higher elevations, with a change in ecosystems that can lead to a decline in populations and a risk of extinction in the near future [13,14]. On the other hand, thermophilic or generalist species can obtain advantages and invade territories that were previously precluded due to temperature limits [15,16]. Alien species, often generalists and with high mobility, can benefit from climate warming [17]. Butterflies are a sensitive taxon to climate changes, and, in mountain areas, specialised species particularly suffer reductions in their distributions and undergo shifts towards higher elevations [18,19]. *Cacyreus marshalli* is a thermophilic species and it could benefit from these changes since its distribution could be limited by low temperatures at high elevations [9]. Given the wide range of factors to consider in relation to the potential impact on *C. marshalli*, it became crucial to obtain deep knowledge of the species, utilising all the available data collected, both according to a standardised survey design and to an opportunistic way. In our study, we applied two different methods of species distribution models (MaxEnt and N-mixture models) to compare different results and approaches in relation to the sampling effort. In the last few years, a huge number of studies have focused on species distribution models [20,21], but less is known about their employment in predicting the expansion of alien species populations and the effect of management activities under different scenarios. The protected areas have the responsibility to maintain their biodiversity heritage and, moreover, to detect and manage early on the expansion of alien species. Collecting data about the presence and invasion of species is essential to predict potential expansion in their territories, to set proper management strategies using a science-based approach and to maximise the probabilities of success in the control of alien species. 

Considering all these aspects, we hypothesized that climate variables could be key drivers of *C. marshalli* distribution and that a temperature increase could exacerbate infestation, even in the colder sites located at high elevations. However, we believe that reducing the number of host plants could mitigate the climate warming effect and lower the risk of potential invasions in new areas. Thus, the aims of our study were to: (1) assess the main drivers of the distribution of the species in Alpine areas; (2) obtain a potential species distribution, comparing different modelling approaches based on different quality datasets (MaxEnt vs. N-mixture models) in order to compare predictions; and (3) evaluate different future scenarios to understand the impact that climate warming may have in the coming years and the possible mitigation effects of management strategies aimed to reduce the presence of the host plant.

## 2. Materials and Methods

### 2.1. Study Area

Our research focused on GPNP, an Alpine protected area in northwest Italy. Its territory extends for 71,043 hectares in two Italian regions (52% of the surface in the Aosta Valley and 48% in Piedmont) and it is mainly mountainous, including the Western Alps. Its elevation ranges between 800 and 4061 m a.s.l. (Gran Paradiso mountain). Thanks to its diversity in terms of habitat types, geological and lithological characteristics, elevation and temperature excursions, the Park hosts a huge amount of biodiversity. 

GPNP includes 37 natural habitat types of community interest, listed in Annex I of the European Commission Habitats Directive (92/43/CEE). More than 1120 floristic species are present, of which 81 are endemics and 6 are protected by the Habitats Directive (Annex II, IV and V). The park is part of the Natura 2000 network and contains 168 vertebrate species (52 mammal, 101 bird, 8 reptile, 3 amphibian and 4 fish species), among which the International Union for Conservation of Nature (IUCN) has classified, at national level, 2 species as critically endangered (CR), 3 species as endangered and 18 as vulnerable (VU). The park has a long tradition of studying invertebrates, mostly butterflies. The park butterfly community includes 121 species, of which 4 are listed in the annexes of the Habitats Directive and are threatened at a European level according to the Red List [6]. Unfortunately, the park is not exempt from alien species invasions. Several alien fish species were introduced in the past for fishing, for example, *Salvelinus fontinalis* Mitchill (1814) (Salmoniformes: Salmonidae), which heavily impacted alpine lake ecosystems and for which the park has launched many projects [22]. Less is known about the presence of alien invertebrates in the protected area. *C. marshalli* is the only alien insect species currently studied, whose presence is recorded inside the park as the focus of a research project undertaken in collaboration with the Zoology Laboratory (ZooLab) of the Department of Life Sciences and Systems Biology (Turin University). 

**Figure 1:**
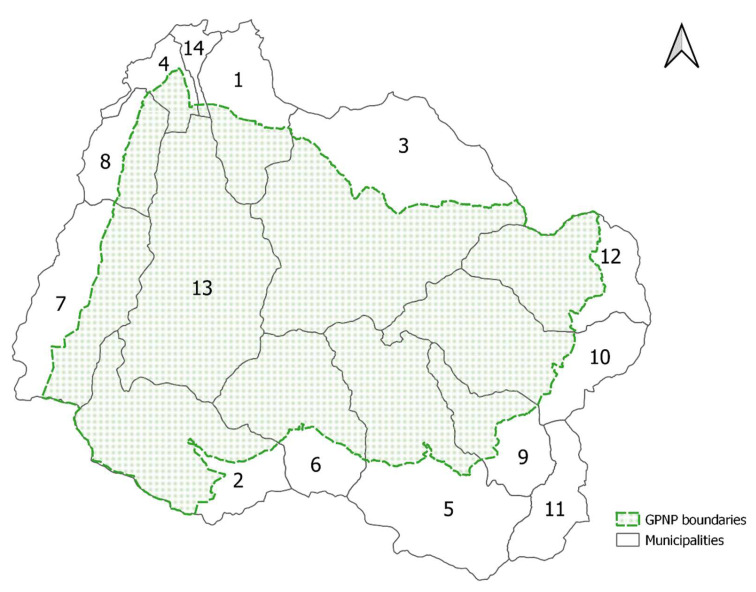
The study area in Gran Paradiso National Park (GPNP). The green dotted line shows the park boundaries that are under the jurisdiction of two regions. Piedmont region: Val Soana (villages: Valprato Soana 12 and Ronco Canavese 10); Valle Orco (villages: Ceresole Reale 2, Noasca, Locana 5, Sparone 11 and Ribordone (9). Aosta Valley region: Valle di Cogne (villages: Cogne 3 and Aymavilles 1); Valsavarenche (villages: Valsavarenche 13, Introd 4 and Villeneuve 14); Val di Rhêmes (villages: Rhêmes-Notre-Dame 7 and Rhêmes-Saint-Georges 8).

The Park’s area includes five mountain valleys split between two Italian regions (two valleys in the Piedmont region and three valleys in the Aosta Valley region). Since most of the urban areas are outside of the protected area, we decided to extend the study area to the bordering municipalities because we considered them operational to study the distribution of *C. marshalli* (Figure 1).

### 2.2. Habitat and Climatic Variables

The habitat and climatic variables were calculated in a grid with a 250 × 250 m resolution covering the entire surface of the study area. We selected seven explanatory variables, two of which were bioclimatic, two related to the host plant and three related to land cover (Table S1): (1) annual mean temperature (*bio01*), (2) temperature seasonality (*bio04*), (3) number of *Pelargonium* pots (*pel_abu*), (4) neighbouring *Pelargonium* abundance (*pel_neigh*), (5) woodland (*wood*), (6) ecotone (*eco*) and (7) grassland (*grass*). We tested the same variables in both analyses using the MaxEnt and N-mixture models.

Annual mean temperature (*bio01*) and temperature seasonality (*bio04*) were extracted from the high-resolution temperature map (250 m spatial resolution) created by Metz et al. [23]. Since the spread of *C. marshalli* is potentially favoured by warm temperatures [9,24], we decided to test the effect of annual mean temperature on butterfly distribution. We then included temperature seasonality in order to better understand how temperature stability or variability among seasons could affect species distribution, probably via influencing phenology and survival at different developmental stages. We did not consider additional climatic variables because the owners of *Pelargonium* usually protect and look after the ornamental plants, balancing the water supply and storing the pots during winter. 

To calculate *pel_abu* and *pel_neigh* variables, we counted the number of ornamental *Pelargonium* pots in all the inside and bordering municipalities of the Gran Paradiso National Park, carrying out the survey in 2017 and following it with two updates in 2018 and 2019. We conducted an exhaustive census during which we counted and georeferenced all the pots containing *Pelargonium* cultivars (see [9] for details). Through the *Pelargonium* census we obtained 247 cells containing *Pelargonium* plants in the study area, with the highest number of 519 pots being located in Cogne Valley. All cells with at least one *Pelargonium* perfectly overlapped the urban environment, confirming the fact that the host plant is unable to spread in nature in our study area. Using the counts of the census, we calculated the number of pelargoniums in the neighbouring cells, considering only the nine cells contiguous to each focus cell. Consideration of food plant abundance is crucial for understanding *C. marshalli* populational trends in relation to the increase in the number of ornamental plants. At the same time, we chose to include in the analysis the number of *Pelargonium* plants in the neighbouring cells to better understand how host plant spatial availability around the focal site (isolated vs. wide availability) could affect ovipositional behaviour and, consequently, *C. marshalli* distribution.

Land cover variables were calculated using a local land cover map developed by the botanical service of the protected area (GIS GPNP Habitat Map 2016—restricted use—www.sit.parco.gran-paradiso.g3wsuite.it (accessed on 1 September 2021)). We obtained, for each cell, land cover percentages for three main habitat categories (woodland, ecotone and grassland) in order to investigate the potential role of macrohabitat structure in determining *C. marshalli* site preferences. The calculation of all the variables was performed with QGIS “Hannover” Version 3.16.11 (2020). 

### 2.3. Cacyreus marshalli Data

#### 2.3.1. Opportunistic Data

We chose to collect opportunistic data (Figure S1) from all the available open access datasets: iNaturalist, Global Biodiversity Information Facility (GBIF) and the national distribution dataset CKmap [25].

We found 138 georeferenced occurrences of *C. marshalli* only in the CkMap dataset. CkMap is a database developed for the publication of distributional data for Italian fauna. It includes an annual updated version of the initial database provided by the Italian Ministry for the Environment [26]. The dataset includes over 210,000 individual records (the 2007 version comprised 60,000 records) mapped on a 10 × 10 km Universal Transverse Mercator (UTM) grid from data available in the scientific literature, from museum collections and from recent reports. Given the fine-grained resolution of our study (250 × 250 m), for our analysis we considered only precisely georeferenced observations (Table S2) and we deleted all occurrences for which only the UTM grid code was known. These observations were of adults, caterpillars and eggs collected from previous studies conducted in the area and from other researchers.

We also considered in the analysis three observations collected by the Gran Paradiso CS project (“Diventa citizen scientist per il Parco”; http://www.pngp.it/en/node/15302 (accessed on 1 September 2021)), in which *C. marshalli* is one of the focal species.

#### 2.3.2. Standardised Sampling Data

In 2018, we collected egg abundance data for *C. marshalli* in the Orco Valley (GPNP) using stratified random sampling. We considered an altitudinal range between 500 and 2000 m and the sampling was based on a grid composed of 3116 cells (250 × 250 m). We grouped our cells in three altitudinal bands: 500–1000 m (band 1), 1000–1500 m (band 2) and 1500–2000 m (band 3). For each cell we calculated the average altitude with QGIS (“Hannover” Version 3.16.11) software using TINITALY DEM, provided by Tarquini et al. [27]. Then, we selected cells that included open areas (meadows, pastures, cultivated areas) covering at least a quarter of the cell’s surface and urban areas (towns and villages). In the higher altitudinal band, for logistical reasons, we only selected cells that included paths to obtain a representative sample for band 3. We defined the sampling sites by randomly selecting 25 cells for each altitudinal band—206, 140 and 220 cells, respectively. Field activities were carried out from July to September to detect eggs during the flight periods of the species. We planned three repetitions for each of the 75 cells on different days to increase the probability of sampling eggs, and we counted the eggs on host plants (ornamental *Pelargonium* spp.). Considering the phenology of the species, we completed the three repetitions within each altitudinal range within a month to count a single generation of the species. Furthermore, the three bands were sampled in a staggered way, starting from the lowest band, in order to follow the different flight periods of *C. marshalli* for each altitudinal band.

### 2.4. Data Analysis

#### 2.4.1. MaxEnt Model

To produce a species distribution model inside the park territories and its neighbouring village using opportunistic data, we used the R package biomod2 [28], employing the algorithm MaxEnt [29]. MaxEnt is a species distribution model based on a max entropy approach that defines the relations between presence distribution points and explanatory variables. This method only analyzes presence data and it is largely used to map species distributions and predict species occurrence correlates; some governmental and non-governmental organizations have adopted MaxEnt as a tool to map biodiversity at large-scale levels (https://www.pointblue.org (accessed on 27 February 2022)). MaxEnt estimates the relative occurrence rate (ROR) or the relative probability that each cell in a study area has a suitable predicted condition for the species in question [30].

It calculates the values of the environmental covariates under species points and compares them with values under background points (pseudo-absence points in which the species’ presence is not assessed). The output of the model predicts the probability of potential distributions [29].

First of all, we randomly selected the pseudo-absence points in a restricted background environment considering a buffer of 2.5 km around the presence points in order to correct the spatial bias occurring in the sampling effort [31]. We chose to create a model including the whole dataset and another using 80% of the data as a training set and the remaining 20% as a test set to evaluate the model following a train–test split procedure. For this last model, we performed 100 replicas in which the training set was chosen randomly for each replica.

#### 2.4.2. N-Mixture Models

We generated N-mixture models to assess *C. marshalli* egg abundance and its relationship with climatic and environmental predictors accounting for imperfect detection. N-mixture models rely on certain key assumptions: closed populations among sampling surveys, no false-positive errors, independence and homogeneity of detectability among individuals.

Since *C. marshalli*’s eggs are easily recognizable and laid uniformly rather than in clustered patterns [8], we can assume that no false positives occurred and that counts were made without a violation of the independence and homogeneity of detection probability assumptions.

In order to respect the closed population assumption, for each altitudinal band we performed egg counts (three sampling repetitions) within 30 days, which corresponded to one flight generation period considering the study area [9]. Moreover, *C. marshalli* larvae do not eat egg chorion, so we would not miss hatched eggs during the counts among sampling surveys. Despite these precautions, adult individuals are mobile animals capable of laying eggs among sampling repetitions, therefore leading to a potential lack of closure. However, N-mixture models could still be an efficient method to evaluate relationships between abundance, climate and environment [32], as it is possible to change the perspective about the estimates of abundance obtained, considering them as the number of individuals (eggs in our case) occurring in the sites during the sampling period rather than as values for absolute egg abundance definitively present in the sites [33]. Therefore, as we could not define the assessed abundances as total egg abundances, we defined them as the numbers of eggs by means of which the level of infestation of each of the sites would be determined, allowing us to assess *C. marshalli* egg distribution over the study area.

Prior to analysis, to avoid collinearity issues, we selected only the explanatory variables showing a Pearson correlation r < |0.7| [34]. We then scaled and centred all the covariates to make them comparable and to facilitate model fitting [35].

Model building was based upon biological hypotheses adding covariates to the null model (*ρ. λ.*) through the *unmarked* package [36] in the statistical software R 4.1.0 (R Core Team, 2021). We applied a two-step modelling approach which consisted of, first, testing predictors for detectability (*ρ*) while keeping abundance (*λ*) constant at null [37]. As we considered only the pelargoniums accessible in each of the sites (*Pelargonium* availability; *pel_ava*) as the variables influencing egg detectability, model testing involved, firstly, a comparison between this simple detection model (*ρ _pel_ava_ λ.*) and the null model (*ρ. λ.*). Once the importance of *Pelargonium* availability had been tested, as a second step, we proceeded with modelling abundance, keeping constant the best model structure for detection probability [38]. Following different biologically relevant combinations, we tested as important predictors of *C. marshalli* egg abundance the following climatic and habitat variables: annual mean temperature (*bio01*), temperature seasonality (*bio04*), *Pelargonium* abundance (*pel_abu*), neighbouring *Pelargonium* abundance (*pel_neigh*), woodland cover (*wood*), ecotone cover (*eco*) and grassland cover (*grass*). Model selection was based on the Akaike information criterion corrected for small sample sizes (AICc), the best model considered to be the one which showed the lowest AICc value and a ΔAICc > 2 compared to the other candidate models [39]. We then focused on a suitable variance structure of the best model by testing and comparing three different distributions for the λ parameter [40]: Poisson (P), negative binomial (NB) and zero-inflated Poisson (ZIP).

To evaluate the predictive ability of the three N-mixture models, we performed a graphical fit assessment by comparing residuals, fitted values and observed data with the *plot.Nmix.resi* function in the R package *AHMbook* [41]. Lastly, we conducted a parametric bootstrap chi-square test of goodness of fit (1000 replicates) for each of the three mixture models using the function *Nmix.gof.test* in the *AICcmodavg* package [42]. Besides the Gof test, the *Nmix.gof.test* function provided a calculation of the overdispersion factor (ĉ), which is important to assess model robustness and, in case of moderate lack of fit [42], to adjust the uncertainties of our estimates [43].

Once all the model diagnostics were performed, we created a distribution map of *C. marshalli* egg abundance over the study area according to the predictions of the best model.

#### 2.4.3. Distribution Maps and Scenarios

Following the outputs of the best species distribution model provided by each approach (MaxEnt vs. N-mixture), we predicted the potential distribution of *C. marshalli* occurrence and egg abundance over the protected area and the bordering municipalities. At first, we created two distribution maps (250 × 250 m cells) representing the predicted values of ROR (as percentages) and the estimated egg abundances of MaxEnt and N-mixture models, respectively. We then looked ahead, mapping *C. marshalli* distribution under a climate warming scenario and applying a management strategy to mitigate a potential climate-induced populational expansion. For the climate warming scenario, we selected a temperature increase of 1.5 °C (RCP 2.6, IPPC 2014) based on the forecast by Gobiet et al. [10] for the year 2050 in the European Alps, and for the mitigation strategy we reduced the host plant species (*Pelargonium* spp.) numbers by 50%, supposing an efficient awareness campaign involving municipalities and citizens. All the maps were created with QGIS software (“Hannover” Version 3.16.11).

## 3. Results

### 3.1. Cacyreus marshalli Data

We collected 138 occurrences in the national dataset CKmap from 2017 to 2019, including the 55 cells that were used for training in the MaxEnt model. Otherwise, we did not find any target species sightings in the two most important CS platforms (iNaturalist and GBIF) for the study area, while only three presence points were found in the park CS project.

Regarding standardised sampling data collection, we counted 685 eggs (in 21 cells) during 225 surveys (sampling repetitions) carried out over 44 days of field work.

### 3.2. MaxEnt Model Results

We obtained high values of TSS for both MaxEnt models (the full model including the whole dataset and the model with the test set)—0.88 and 0.87, respectively.

**Figure 2:**
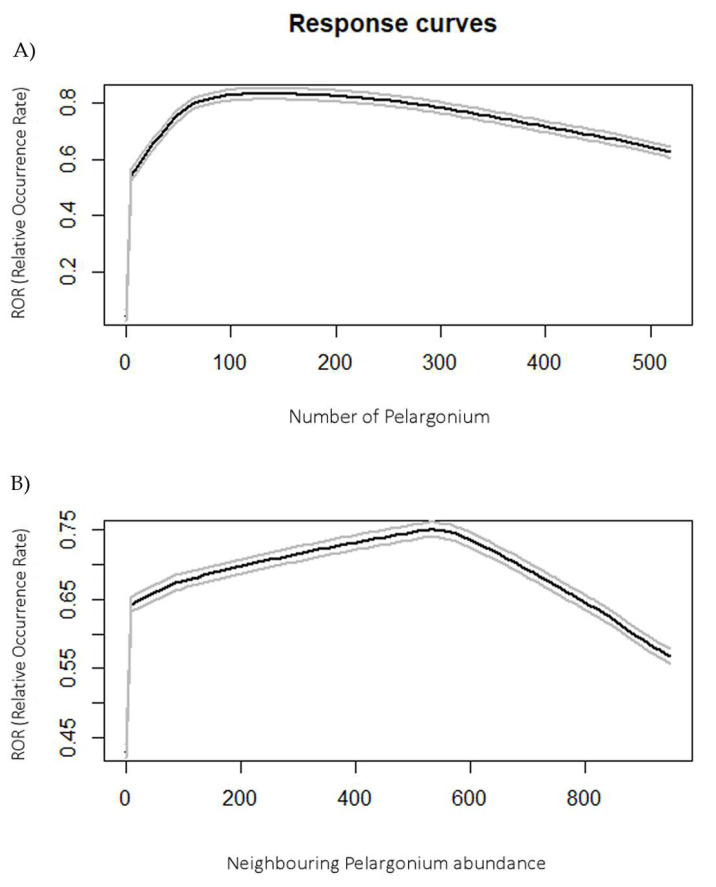
The graphs show the response curves (black lines) obtained with the MaxEnt model, including the confidence intervals (grey lines). (**A**) Along the x-axis: the number of *Pelargonium* plants; along the y-axis: the relative occurrence rate (ROR). (**B**) Along the x-axis: the number of *Pelargonium* plants in the neighbouring cells; along the y-axis: the ROR.

*Pel_abu* was found to be the most important variable in the models, with a percentage contribution of 90% in the full model and 88% in the second model (Table 1). Indeed, the cells with high ROR values overlapped perfectly the cells containing pelargoniums (Figure 2). The *pel_negh* variable played a marginal role in the model, considering the contribution of 7.2% in the full model and 9.7% in the model using a test set. The variables regarding temperature data (*bio01*, *bio04*) did not make a relevant contribution to the models, showing percentage contributions of less than 2% and 0.5% for temperature seasonality and annual mean temperature, respectively. Habitat variables (woodland, ecotone and grassland) proved not to be significant variables (contributions < 0.01%); thus, we did not report them in Table 1.

Thanks to the MaxEnt algorithm, we obtained the response curves for each variable. ROR values rapidly increased, even with a low number of food plants in the cell (Figure 2). At the same time, ROR value decreased in the cells when the number of neighbouring plants was higher than 600 units (Figure 2). Due to the insignificance of the climatic variables, we chose to not report them.

The MaxEnt analysis did not provide relevant ecological information about *C. marshalli* distribution. Indeed, the variables that made the most important contributions were linked to the host plant (Table 1), as we could have expected, since species presence is obviously dependent on *Pelargonium*. Therefore, we did not run the scenario models using the MaxEnt algorithm.

### 3.3. N-Mixture Model Results

**Figure 3:**
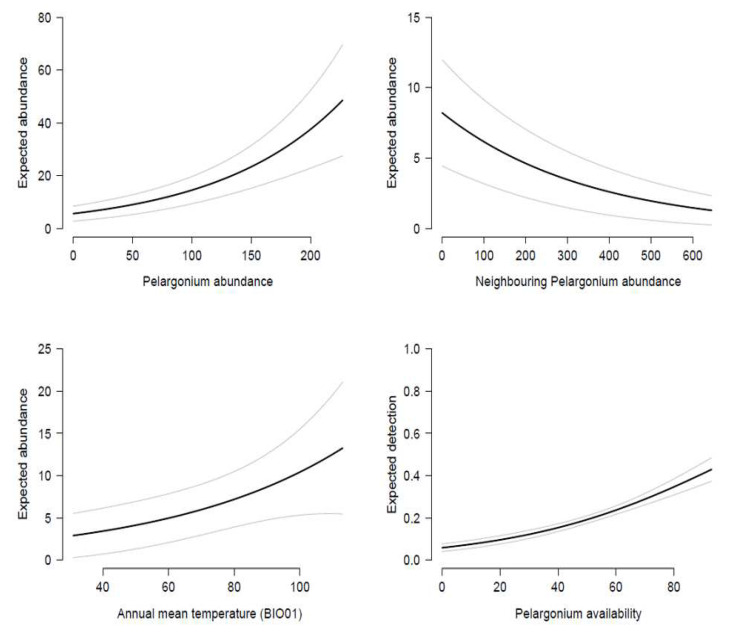
Single covariate responses in relation to the expected abundance and detection predicted by the best N-mixture model. Grey lines indicate 1-SE bounds.

The first step of our modelling approach highlighted, as we supposed, the importance of the *pel_ava* variable for detecting *C. marshalli* eggs (Figure 3). Indeed, looking at model selection, we noticed that the detection model *ρ _pel_ava_ λ.* showed a definitely higher AICc value than the null model (ΔAIC_c_ = 1089.19; Table 2).

Once the observation process structure had been defined, we focused on testing the predictors for the state process. Firstly, we observed that *bio01*, *pel_abu* and *pel_neigh* were the most important variables affecting egg abundance (Table 2). We then hypothesised a potential synergistic action between the *bio01* and *pel_abu* variables; thus, we added to the previous best model (*ρ _pel_ava_ λ _bio01 + pel_abu + pel_neigh_*; Table 2) an interaction term. The last model structure (*ρ _pel_ava_ λ _bio01 + pel_abu + pel_abu:bio01 + pel_neigh_*) definitely proved to be the best one (Table 2), highlighting positive effects of *bio01*, *pel_abu* and the interaction term *pel_abu: bio01* on egg abundance, while *pel_neigh* was shown to have a negative effect (Table 3). The model’s output showed that a rise in temperature and host plant abundance favoured egg abundance, while an increase in neighbouring *Pelargonium* availability resulted in a decreasing number of eggs (Figure 3). The positive synergistic action between *bio01* and *pel_abu* revealed that the more temperature increases, the more *Pelargonium* abundance positively affects *C. marshalli* egg abundance.

Once we had identified the best N-mixture model, we tested the best mixture for abundance comparing P, NB and ZIP distributions. Model selection found NB to have the best λ distribution (ΔAIC_c_ = 97.43), while ZIP and P showed the second and third AIC_c_ values, respectively (Table S3). Since NB distribution is generally favoured by AIC_c_ selection [41] and it could sometimes provide higher abundance estimates [32,44,45], we examined the residuals, fitted values and observed data of the three models. The graphical fit assessment highlighted that the ZIP model had a much better predictive ability than the NB and P models (for details, see Figure S2). Moreover, the ZIP model was the only model to pass the goodness of fit test for chi-squared statistics (*p* = 0.06) and, considering the many zeros characterising our egg counts (zeros = 80%), we felt confident in selecting the ZIP mixture as a suitable distribution for abundance. Although the ZIP model adequately fitted the data, the goodness of fit test showed weak model robustness, as highlighted by the c-hat parameter (ĉ = 2.68), too. We therefore used calculated overdispersion (OD) to multiply the variance–covariance matrix of the ZIP model in order to inflate the uncertainties of our estimates according to an OD factor.

### 3.4. Distribution Maps and Scenario Results

**Figure 4:**
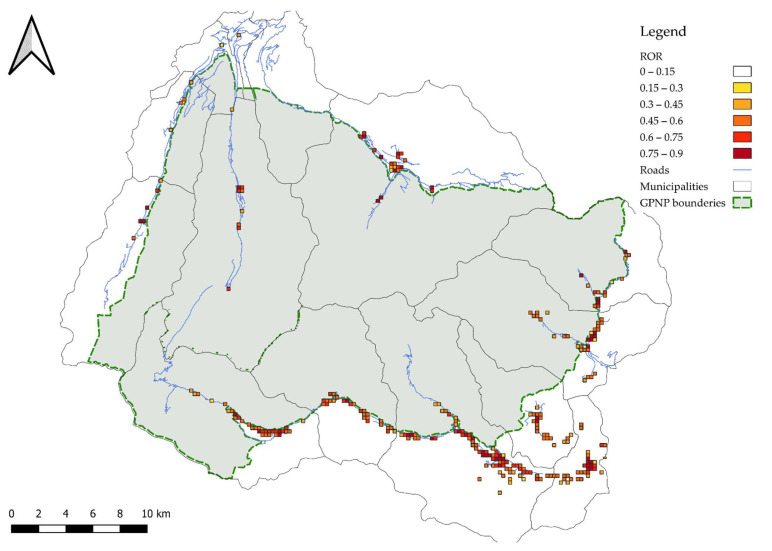
The map shows the output of the MaxEnt model ROR, or the relative probability that each has a suitable predicted condition for *Cacyreus marshalli*. The borders of the municipalities included in the analysis are shown in green, while the streets are in grey. The grey polygon marks the Park territory, in which only one sampled valley is included entirely. The colour scale, in the top right corner, indicates the ROR values.

The MaxEnt map highlighted that most of the cells showed high ROR values, indicating a wide potential distribution of *C. marshalli* over the study area considered (Figure 4). This result is not surprising since the host plant is present in all the cells and even low *Pelargonium* abundance determines a high percentage of site suitability (ROR = 64%).

**Figure 5:**
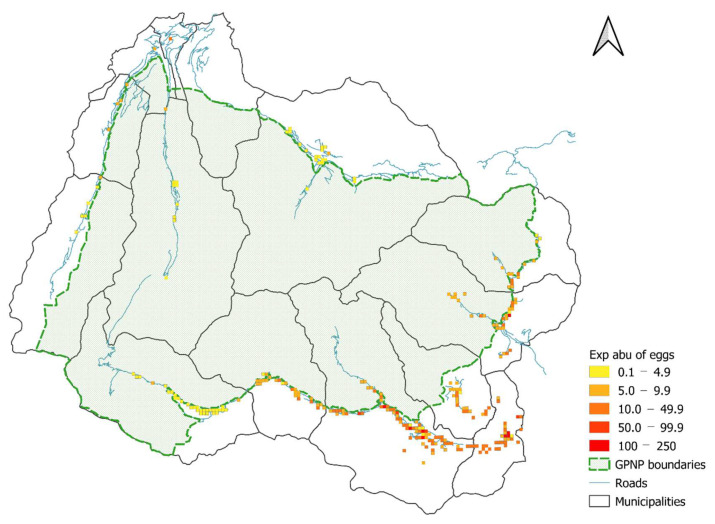
Expected abundance of eggs in the study area (250 m grid) obtained from the best N-mix model, ρ *_pel_ava_*λ *_bio01 + pel_abu + pel_abu:bio01 + pel_neigh_*.

Concerning N-mixture model projections, we noticed a high level of infestation in the low- and middle-elevation municipalities, while we found low egg abundances in cells overlapping the high-altitude municipalities in both regions (Figure 5). The Aosta Valley side of GPNP showed an overall low rate of egg infestation compared to the Piedmont region (Figure 5).

**Figure 6:**
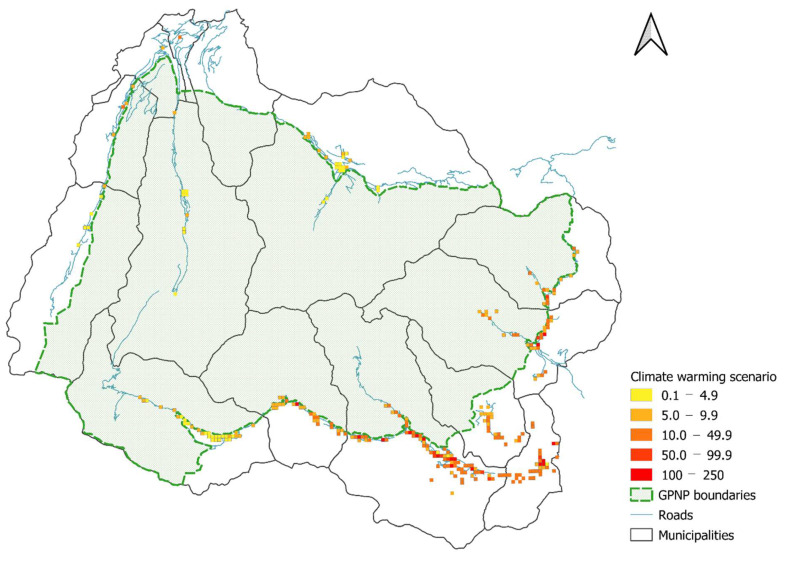
Expected abundance of eggs in the study area (250 m grid) in the future scenario of climate warming (increase of 1.5 °C).

**Figure 7:**
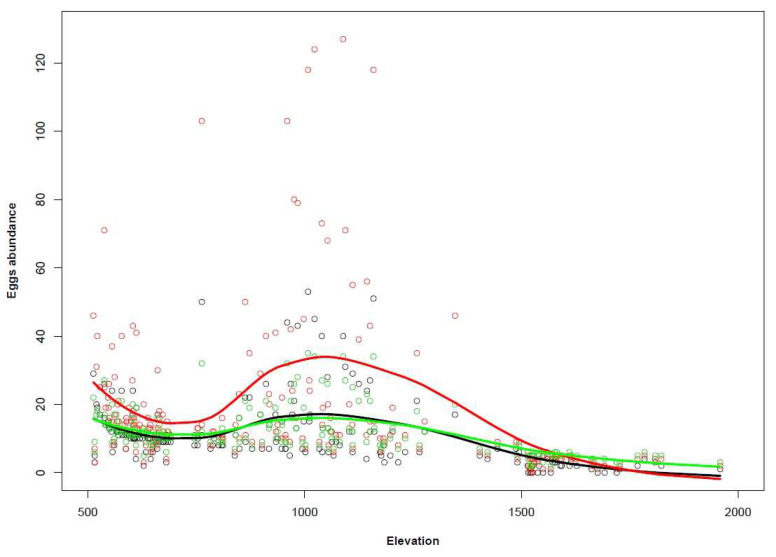
Scatter plot with three trend lines (*loess*) showing egg abundance distributions in relation to elevation for the three scenarios. The black line represents the initial potential distribution, the red line the climate warming scenario (+1.5 °C) and the green line the climate warming scenario combined with management action (50% reduction in *Pelargonium* plants). Extreme values (*n* = 250) were omitted to improve the graphical representation.

**Figure 8:**
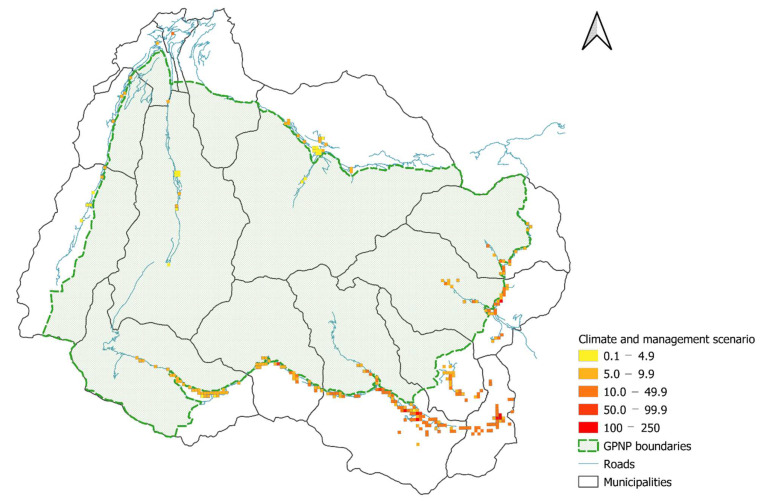
Expected abundance of eggs in the study area (250 m grid) in the future scenario of climate warming (increase of 1.5 °C) combined with the management strategy (50% reduction in *Pelargonium* plants).

The potential rise in temperatures (1.5 °C) revealed that nearly all the cells experienced increases in egg abundance (Table 4; Figure 6) along the whole elevational gradient, while there were some exceptions at high elevations (Figure 7). Only a few cells showed new egg infestations (Table 4), but all the newly infested sites were located in high-altitude municipalities (Rhêmes-Notre-Dame and Cogne). We then tested the climate change mitigation strategy (50% Pelargonium reduction) and we noticed that the hypothetical management action affected egg abundance in most of the cells (Table 4; Figure 8), with a significant egg reduction in the low- and middle-elevation municipalities (Table 5). However, the changes observed were not restricted solely to egg decreases but, surprisingly, also to slight increases (Table 4; Figure 7), which were restricted mainly to high-elevation sites (Table 5). Generally, the 50% Pelargonium reduction restored *C. marshalli* egg distribution to the pre-temperature increase scenario, with a slight signal of a countertrend in the high-elevation municipalities (Table 5; Figure 7). Considering the temperature elevation relationship (higher elevation–colder temperatures; [46]), we reported the results of the distribution maps and the scenarios following municipalities and elevation because we considered them easily interpretable and more informative for management planning.

## 4. Discussion

### 4.1. Opportunistic Data and Standardised Sampling

Our research and the relative statistical analyses performed (MaxEnt vs. N-mixture models) highlighted different results obtained with the two approaches (opportunistic vs. standardised sampling). The comparison between methods revealed how N-mixture analysis, supported by a standardised sampling design, provided much more relevant ecological information compared to MaxEnt. This difference could have been caused by the minor sampling effort which characterised the opportunistic data collection [47]. Indeed, we found 141 *C. marshalli* presence points, the majority of which came from the national dataset (CKmap and integration from Balletto and colleagues), with only three sightings coming from the Park’s CS project. Therefore, we noted some difficulties with respect to citizens’ reports of species occurrence, probably due to the fact that the focal species is not particularly conspicuous and so not as easy for local people to detect [48]. *C. marshalli* is a small and cryptic butterfly [4]. The larvae exhibit mimetic behaviour and imitate *Pelargonium* stem colours; the adult is tiny and brown and, because of its erratic flight pattern and the variety of nectar feeding sources, it is difficult to observe and photograph. To obtain a large number of observations, it is important to improve citizen scientists’ engagement by increasing training activities with the fundamental support of the Park [49]. CS data have become more and more important in the last few years, and they have largely been employed in species distribution models [50]. Furthermore, among CS projects, butterflies are often used as target groups because they are easily recognisable at the species level, well known and highly charming for the general public [51]. Indeed, nowadays, butterflies are the only group of invertebrates that are monitored at the European level through the involvement of citizens in a standardised monitoring scheme (see the European Butterfly Monitoring Scheme). Projects run by local institutions are mostly designed to obtain opportunistic observations, which have great potential use value in modelling approaches. However, without an efficient communication campaign, these projects generally collect few and narrow data with an unknown sampling effort [47], while, in order to obtain relevant ecological information, it is fundamental to obtain a high number of observations over vast territories [52]. A significant difference between opportunistic and standardised sampling is surely the sampling effort, but it is fundamental to consider the role of sampling design, too. The N-mixture analysis provided much more relevant ecological information due to the stratified random sampling design adopted, which allowed us to investigate an equal number of sites over different altitudinal bands characterised by heterogeneous temperature regimes. Such site variability, in harmony with the N-mixture analysis, revealed the important relationship between temperature, host plant presence and egg infestation, underlining the important role of a planned survey design in investigations of species habitat relationships and their distributions. Obviously, a rigorous survey protocol needs expert researchers, logistical support, time and a large amount of resources [53], often covering a limited spatial extent, nevertheless.

For these reasons, we argue that the truth lies in the middle, so an integrated approach that combines opportunistic data with counts from a designed protocol could be the way [54] to optimise the amount of information achievable with sustainable efforts and resources.

### 4.2. Drivers of Cacyreus marshalli Distribution 

The MaxEnt analysis mainly suggested that urban areas are suitable habitats for *Cacyreus marshalli* because of the considerable presence of the food plant (*Pelargonium*) widely used as an ornamental plant in the municipalities inside and bordering the Park.

Our results indicate that the species prefers its native host plant; when *Pelargonium* is available, the exotic butterfly is not driven to naturalise itself on spontaneous food sources such as native Geraniaceae, although it could have the potential to do so [8,9]. An analogous relationship between urban habitats and distribution was found for another alien insect, the Asian tiger mosquito (*Aedes albopictus* (Skuse, 1894) (Diptera: Culicidae)). This species has rapidly adapted to the newly invaded areas but has never been observed using spontaneous trees as oviposition sites because it prefers sub-pots or tires that are typical cavities in the urban areas [55]. The strong connection between *C. marshalli* and its food plant was confirmed by the high contribution of the *pel_abu* variable in explaining both *C. marshalli* occurrence and egg abundance, even with low levels of *Pelargonium* abundance. The host plant demonstrated its importance in shaping *C. marshalli* distribution, also, in terms of spatial availability. Indeed, the more *Pelargonium* abundance increases in neighbouring cells, the less the alien butterfly lays its eggs in focal cells. This negative relation could be explained by the propensity of the females to spread their eggs on different plants due to the strong territorial behaviour of offspring [4]. Therefore, when the host plant is widely distributed, the number of eggs in a site tends to decrease as they are evenly propagated.

As *C. marshalli* is a species native to southern Africa, we hypothesised that temperature could play an important role in driving species distributions, as suggested by Paradiso et al. [9]. N-mixture models supported our hypothesis, highlighting a clear preference of *C. marshalli* for mild temperatures, while, contrarily, it suffers in cold temperatures.

With annual mean temperatures below 4 °C, egg abundance shows very low values. Thus, we can definitely assert that cold temperatures constrain butterfly distribution. For insect groups, it is generally recognized that temperatures are important with respect to limiting or facilitating the invasion process [56], and *C. marshalli* is not an exception. The importance of temperature in limiting *C. marshalli* distribution is underlined, furthermore, by the positive interactive effect with *Pelargonium* abundance. Temperature regulates the effect of the host plant variable which is weaker in colder sites than in warmer ones. Indeed, with an equal number of *Pelargonium* plants, the areas showing high temperatures are definitely more heavily infested than areas with low temperatures.

### 4.3. Distribution Maps, Future Scenarios and a Potential Management Strategy

The potential distribution of *C. marshalli* obtained by the N-mixture model analysis showed that high-elevation areas present low levels of egg infestation. This is largely because the high-elevation sites are characterised by cold temperatures, so that egg abundance is consequently limited. For the same reason, in the Aosta Valley region we noticed a lower number of eggs compared to the Piedmont region. Indeed, most of the Aosta Valley municipalities are located in a territory with a mean elevation higher than Piedmont (see Table 5), and therefore cold temperatures, typical of high elevations, constrain egg abundance.

The role of temperature in shaping *C. marshalli* distribution is also supported by the temperature increase scenario. A 1.5 °C increase in temperature positively affected egg abundance over the whole study area, with a slight signal of expansion even in some high-elevation sites. Climate change is a crucial driving factor in the invasion process of alien species [57]. In particular, warming temperatures are strictly related to the expansion and establishment of exotic insect species [58]. Our research is in accordance with this statement. A potential temperature rise would increase the establishment rate of *C. marshalli* and favour a weak expansion trend in areas at high elevations that, due to the temperature increase, would become more suitable as habitats.

To overcome the consequences of climate warming on *C. marshalli* distribution, we considered a 50% *Pelargonium* reduction which resulted in a mitigation of egg infestation in most of the sites located at low and middle elevations. The tested management action demonstrated that it could be possible to reduce the climate warming effect in the areas mainly affected by high rates of infestation, resulting in an egg distribution similar to the pre-temperature increase scenario. However, focusing on high-elevation areas, we noticed an opposite trend which indicates that a *Pelargonium* containment would result in an increase in egg abundance, even though only very slight. This countertrend could be likely caused by low *Pelargonium* availability on a wide spatial scale (host plants in neighbouring cells) in high-elevation sites which are less likely to have urban characteristics. Indeed, a further reduction in *Pelargonium* numbers could diminish the negative effect of *pel_neigh* on egg abundance, which therefore could lead to increases in focal cells. A potential increase in egg numbers in a focal cell may lead to a saturation process which could stimulate the alien butterfly to explore new adjacent sites where native host plants are reduced or absent and thus lay eggs on native Geraniaceae. The potential naturalisation risk could be exacerbated by the integration of high-elevations cells in a matrix of proximal natural habitats.

We therefore face a paradoxical effect caused by the management action which, acting on the abundance and the availability of such a vital requirement as the host plant, should have theoretically reduced the rate of infestation over the whole study area. According to this result, we argue that it is important to previously test management actions designed to control alien species because they could be counterproductive. However, although we have highlighted these contrasting signals, we suggest being cautious in interpreting these results because the changes in egg abundances in high-elevation sites are really slight.

## 5. Conclusions

In conclusion, the field survey protocol proved more informative than the opportunistic data collection because of the higher sampling effort and the planned sampling design. However, opportunistic data derived from CS projects could have a great potential if they are supported by investments and efficient communication campaigns. In this framework, protected areas could play a crucial role in organizing and promoting CS activities combined with research projects according to an integrated approach.

Temperature is a key driver in the establishment, expansion and restriction of *C. marshalli*. The modelling results showed that high-elevation areas were generally protected by low temperatures, even if temperatures increased by 1.5 °C and the host plant was abundant, while low- and middle-elevation sites showed a dramatic increase in egg infestation rates under the climate warming scenario. We suggest that awareness ought to be raised of the risks associated with a potential higher temperature scenario (>1.5 °C). A 50% reduction in host plant numbers could mitigate the effect of the rising temperatures, but at high elevations we noticed a slight countertrend. We therefore urge that management actions not be applied without first testing the effects in the local territorial context.

## Figures and Tables

**Table 1:** The table shows the importance of each variable for the full MaxEnt model (including all the data) but also the mean and the standard error for the 100 interactions tested on the split dataset that included 80% of the data and 20% used as a test set: annual mean temperature (*bio01*), temperature seasonality (*bio04*), *Pelargonium* abundance (*pel_abu*), neighbouring *Pelargonium* abundance (*pel_neigh*).

Variables	Full Model		Test Set Model	
	Percent Contribution	Permutation Importance	Percent Contribution	Permutation Importance
*pel_abu*	90.7	70.7	88.1	74.8
*pel_neigh*	7.2	6.3	9.7	8.6
*bio04*	1.9	22.7	1.8	15.7
*bio01*	0.1	0.3	0.4	0.8

**Table 2:** Model selection of N-mixture models for *Cacyreus marshalli* egg abundance. Annual mean temperature (*bio01*), temperature seasonality (*bio04*), *Pelargonium* abundance (*pel_abu*), neighbouring *Pelargonium* abundance (*pel_neigh*), woodland cover (*wood*), ecotone cover (*eco*) and grassland cover (*grass*). K = number of parameters, AICc = Akaike information criterion corrected for small samples, ΔAICc = AICc difference, Wi = weight of each model, Cum.Wi = cumulative weight.

Models	K	AICc	ΔAICc	W_i_	Cum.W_i_
*ρ _pel_ava_ λ _bio01 + pel_abu + pel_abu:bio01 + pel_neigh_*	7	979.54	0.00	1	1
*ρ _pel_ava_ λ _bio01 + pel_abu + pel_neigh_*	6	1099.16	119.61	<0.01	1
*ρ _pel_ava_ λ _bio01 + pel_abu_*	5	1102.05	122.51	<0.01	1
*ρ _pel_ava_ λ _bio01 + pel_abu + wood_*	6	1102.58	123.04	<0.01	1
*ρ _pel_ava_ λ _bio01_*	4	1135.79	156.24	<0.01	1
*ρ _pel_ava_ λ _bio01 + pel_neigh_*	5	1136.25	156.70	<0.01	1
*ρ _pel_ava_ λ _bio01 + wood_*	5	1138.09	158.54	<0.01	1
*ρ _pel_ava_ λ _bio04_*	4	1186.08	206.53	<0.01	1
*ρ _pel_ava_ λ _pel_neigh_*	4	1273.59	294.05	<0.01	1
*ρ _pel_ava_ λ _wood_*	4	1275.42	295.88	<0.01	1
*ρ _pel_ava_ λ _eco_*	4	1281.87	302.33	<0.01	1
*ρ _pel_ava_ λ _eco_*	4	1282.99	303.45	<0.01	1
*ρ _pel_ava_ λ.*	3	1286.70	307.15	<0.01	1
*ρ _pel_ava_ λ _grassland_*	4	1287.91	308.36	<0.01	1
*ρ. λ.*	2	2375.88	1396.33	<0.01	1

**Table 3:** *β* estimates for the best fitting N-mixture model.

Variables	Egg Abundance (SE)	Detectability (SE)
Intercept	3.095 (0.249) **	−2.632 (0.182) **
*bio01*	0.446 (0.209) *	
*pel_abu*	0.320 (0.058) **	
*pel_neigh*	−0.343 (0.067) **	
*pel_abu:bio01*	0.592 (0.119) **	
*pel_ava*		0.399 (0.049) **

* *p* value < 0.05; ** *p* value < 0.01.

**Table 4:** Percentages of cells showing changes in egg abundance under different scenarios.

Change Categories	Starting Distribution vs. +1.5 °C Scenario	+1.5 °C Scenario vs. −50% *Pelargonium* Scenario
Percentage of cells with egg abundance changes	98.1 (264)	74 (199)
Percentage of cells with egg increases	98.1 (264)	22.7 (61)
Percentage of cells with egg decreases	0	51.3 (138)
Percentage of cells with new egg infestations	3.3 (9)	0
Percentage of cell with no changes	5.2 (14)	29.7 (80)

**Table 5:** Percentages of cells that showed a decrease or increase in egg abundance once the 50% *Pelargonium* reduction was applied.

Municipality	Elevation	Percentage of Cells with Egg Decreases	Percentage of Cells with Egg Increases
Sparone	614	72.7 (24)	9.1 (3)
Locana	714	70.5 (55)	15.4 (12)
Villeneuve	966	100 (1)	0
Ronco Canavese	1050	56.7 (17)	3.3 (1)
Introd	1063	0	0
Noasca	1071	53.8 (14)	11.5 (3)
Ribordone	1127	52.2 (12)	8.7 (2)
Rhêmes-Saint-Georges	1249	75 (3)	0
Valprato Soana	1268	66.7(10)	13.3 (2)
Ceresole Reale	1581	0	40.6 (13)
Cogne	1586	9.5 (2)	66.7 (14)
Valsavarenche	1635	0	75 (6)
Rhêmes-Notre-Dame	1699	0	83.3 (5)

## Data Availability

Not applicable.

## Supplementary Materials

The following are available online at https://www.mdpi.com/article/10.3390/biology11040563/s1, Table S1: Summary of the explanatory variables used for MaxEnt and N-mixture analysis, Table S2: Details on the occurrence data used for Maxent analysis, Table S3: Model selection of the selected N-mixture model with different distributions for the λ parameter, Figure S1: *Cacyreus Marshall* presence points, Figure S2: Model residual diagnostics for the N-mixture models.

## References

[1] Walther G.-R., Roques A., Hulme P.E., Sykes M.T., Pyšek P., Kühn I., Zobel M., Bacher S., Botta-Dukat Z., Bugmann H. (2009). Alien species in a warmer world: Risks and opportunities. Trends Ecol. Evol..

[2] Bellard C., Cassey P., Blackburn T.M. (2016). Alien species as a driver of recent extinctions. Biol. Lett..

[3] Pyšek P., Hulme P.E., Simberloff D., Bacher S., Blackburn T.M., Carlton J.T., Dawson W., Essl F., Foxcroft L.C., Genovesi P. (2020). Scientists’ warning on invasive alien species. Biol. Rev..

[4] Clark G.C., Dickson C.G.C. (1971). Life Histories of the South African Lycaenid Butterflies.

[5] Dukes J.S., Mooney H.A. (1999). Does global change increase the success of biological invaders?. Trends Ecol. Evol..

[6] Van Swaay C.C., Cuttelod A., Collins S., Maes D., Munguira M.L., Šašić M., Settele J., Verovnik R., Verstrael T., Warren M. (2010). European Red List of Butterflies.

[7] Sarto i Monteys V. (1992). Spread of the southern African lycaenid butterfly, *Cacyreus marshalli* Butler, 1898, (Lep.: Lycaenidae) in the Balearic Archipelago (Spain) and considerations on its likely introduction to continental Europe. J. Res. Lepid..

[8] Quacchia A., Ferracini C., Bonelli S., Balletto E., Alma A. (2008). Can the Geranium Bronze, *Cacyreus marshalli*, become a threat for European biodiversity?. Biodivers. Conserv..

[9] Federica P., Francesca M., Cristiana C., Silvia G., Ramona V., Sara C., Chiara F., Simona B. (2019). From Africa to the Alps: Risk assessment on an invasion by *Cacyreus marshalli* (Butler, 1898). J. Insect Conserv..

[10] Gobiet A., Kotlarski S., Beniston M., Heinrich G., Rajczak J., Stoffel M. (2014). 21st century climate change in the European Alps—A review. Sci. Total Environ..

[11] Parolo G., Rossi G. (2008). Upward migration of vascular plants following a climate warming trend in the Alps. Basic Appl. Ecol..

[12] Brivio F., Zurmühl M., Grignolio S., von Hardenberg J., Apollonio M., Ciuti S. (2019). Forecasting the response to global warming in a heat-sensitive species. Sci. Rep..

[13] Jacobson A.R., Provenzale A., von Hardenberg A., Bassano B., Festa-Bianchet M. (2004). Climate forcing and density dependence in a mountain ungulate population. Ecology.

[14] Rogora M., Frate L., Carranza M., Freppaz M., Stanisci A., Bertani I., Bottarin R., Brambilla A., Canullo R., Carbognani M. (2018). Assessment of climate change effects on mountain ecosystems through a cross-site analysis in the Alps and Apennines. Sci. Total Environ..

[15] Gottfried M., Pauli H., Futschik A., Akhalkatsi M., Barančok P., Benito Alonso J.L., Coldea G., Dick J., Erschbamer B., Calzado F. (2012). Continent-wide response of mountain vegetation to climate change. Nat. Clim. Chang..

[16] Fumy F., Löffler F., Samways M.J., Fartmann T. (2020). Response of Orthoptera assemblages to environmental change in a low-mountain range differs among grassland types. J. Environ. Manag..

[17] Poniatowski D., Beckmann C., Löffler F., Münsch T., Helbing F., Samways M.J., Fartmann T. (2020). Relative impacts of land-use and climate change on grasshopper range shifts have changed over time. Glob. Ecol. Biogeogr..

[18] Cerrato C., Rocchia E., Brunetti M., Bionda R., Bassano B., Provenzale A., Bonelli S., Viterbi R. (2019). Butterfly distribution along altitudinal gradients: Temporal changes over a short time period. Nat. Conserv..

[19] Rödder D., Schmitt T., Gros P., Ulrich W., Habel J.C. (2021). Climate change drives mountain butterflies towards the summits. Sci. Rep..

[20] Westwood R., Westwood A.R., Hooshmandi M., Pearson K., LaFrance K., Murray C. (2020). A field-validated species distribution model to support management of the critically endangered Poweshiek skipperling (*Oarisma poweshiek*) butterfly in Canada. Conserv. Sci. Pract..

[21] Casazza G., Abeli T., Bacchetta G., Dagnino D., Fenu G., Gargano D., Minuto L., Montagnani C., Orsenigo S., Peruzzi L. (2021). Combining conservation status and species distribution models for planning assisted colonisation under climate change. J. Ecol..

[22] Tiberti R., Nelli L., Brighenti S., Iacobuzio R., Rolla M. (2017). Spatial distribution of introduced brook trout *Salvelinus fontinalis* (Salmonidae) within alpine lakes: Evidences from a fish eradication campaign. Eur. Zool. J..

[23] Metz M., Rocchini D., Neteler M. (2014). Surface temperatures at the continental scale: Tracking changes with remote sensing at unprecedented detail. Remote Sens..

[24] Kučinić M., Koren T., Mihoci I., Vuković M., Bukovec D., Jakovljević T., Jenčić S. (2013). Can spreading of the Geranium Bronze *Cacyreus marshalli* (Butler, 1898)(Insecta, Lepidoptera, Lycaenidae) in Croatia be assigned to climate change?. Period. Biol..

[25] Ruffo S. (2005). Checklist e Distribuzione della Fauna Italiana 10,000 Specie Terrestri e Delle Acque Interne.

[26] Balletto E., Bonelli S., Cassulo L., Ruffo S., Stoch F. (2007). Insecta Lepidoptera Papilionoidea. Checklist and Distribution of the Italian Fauna 10,000 Terrestrial and Inland Water Species.

[27] Tarquini S., Nannipieri L. (2017). The 10 m-resolution TINITALY DEM as a trans-disciplinary basis for the analysis of the Italian territory: Current trends and new perspectives. Geomorphology.

[28] Thuiller W., Georges D., Engler R., Breiner F., Georges M.D., Thuiller C.W. (2016). Ensemble Platform for Species Distribution Modeling. Package ‘biomod2’. Species Distribution Modeling Within an Ensemble Forecasting Framework; version 3.3-7. Ecography.

[29] Phillips S.J., Anderson R.P., Schapire R.E. (2006). Maximum entropy modeling of species geographic distributions. Ecol. Model..

[30] Merow C., Smith M.J., Silander J.A. (2013). A practical guide to MaxEnt for modeling species’ distributions: What it does, and why inputs and settings matter. Ecography.

[31] Fourcade Y., Engler J.O., Rödder D., Secondi J. (2014). Mapping species distributions with MAXENT using a geographically biased sample of presence data: A performance assessment of methods for correcting sampling bias. PLoS ONE.

[32] Joseph L.N., Elkin C., Martin T.G., Possingham H.P. (2009). Modeling abundance using N-mixture models: The importance of considering ecological mechanisms. Ecol. Appl..

[33] Kéry M., Royle J.A. (2015). Applied Hierarchical Modeling in Ecology: Analysis of Distribution, Abundance and Species Richness in R and BUGS.

[34] Dormann C.F., Elith J., Bacher S., Buchmann C., Carl G., Carré G., Marquéz J.R.G., Gruber B., Lafourcade B., Leitão P.J. (2013). Collinearity: A review of methods to deal with it and a simulation study evaluating their performance. Ecography.

[35] Becker H., Leon J. (1988). Stability analysis in plant breeding. Plant Breed..

[36] Fiske I., Chandler R. (2011). Unmarked: An R package for fitting hierarchical models of wildlife occurrence and abundance. J. Stat. Softw..

[37] Porro Z., Odicino M., Bogliani G., Chiatante G. (2021). Intensive forestry and biodiversity: Use of poplar plantations by woodpeckers in a lowland area of Northern Italy. For. Ecol. Manag..

[38] Kroll A.J., Fleming T.L., Irwin L.L. (2010). Site occupancy dynamics of northern spotted owls in the eastern Cascades, Washington, USA, 1990–2003. J. Wildl. Manag..

[39] Burnham K.P., Anderson D.R. (2004). Multimodel inference: Understanding AIC and BIC in model selection. Sociol. Methods Res..

[40] Wenger S.J., Freeman M.C. (2008). Estimating species occurrence, abundance, and detection probability using zero-inflated distributions. Ecology.

[41] Kéry M., Royle A., Meredith M. (2017). AHMbook: Functions and Data for the Book ‘Applied Hierarchical Modeling in Ecology’. https://CRAN.R-project.org/package=AHMbook.

[42] Mazerolle M. (2015). AICcmodavg: Model selection and multimodel inference based on (Q) AIC (c). https://cran.r-project.org/package=AICcmodavg.

[43] Johnson D.S., Laake J.L., Ver Hoef J.M. (2010). A model-based approach for making ecological inference from distance sampling data. Biometrics.

[44] Kéry M., Royle J.A., Schmid H. (2005). Modeling avian abundance from replicated counts using binomial mixture models. Ecol. Appl..

[45] Ver Hoef J.M., Boveng P.L. (2007). Quasi-Poisson vs. negative binomial regression: How should we model overdispersed count data?. Ecology.

[46] Rolland C. (2003). Spatial and seasonal variations of air temperature lapse rates in Alpine regions. J. Clim..

[47] Dickinson J.L., Zuckerberg B., Bonter D.N. (2010). Citizen science as an ecological research tool: Challenges and benefits. Annu. Rev. Ecol. Evol. Syst..

[48] McKinley D.C., Miller-Rushing A.J., Ballard H.L., Bonney R., Brown H., Cook-Patton S.C., Evans D.M., French R.A., Parrish J.K., Phillips T.B. (2017). Citizen science can improve conservation science, natural resource management, and environmental protection. Biol. Conserv..

[49] Martin V.Y. (2017). Citizen science as a means for increasing public engagement in science: Presumption or possibility?. Sci. Commun..

[50] Feldman M.J., Imbeau L., Marchand P., Mazerolle M.J., Darveau M., Fenton N.J. (2021). Trends and gaps in the use of citizen science derived data as input for species distribution models: A quantitative review. PLoS ONE.

[51] Lewandowski E.J., Oberhauser K.S. (2017). Butterfly citizen scientists in the United States increase their engagement in conservation. Biol. Conserv..

[52] Dickinson J.L., Shirk J., Bonter D., Bonney R., Crain R.L., Martin J., Phillips T., Purcell K. (2012). The current state of citizen science as a tool for ecological research and public engagement. Front. Ecol. Environ..

[53] Pacifici K., Reich B.J., Miller D.A., Gardner B., Stauffer G., Singh S., McKerrow A., Collazo J.A. (2017). Integrating multiple data sources in species distribution modeling: A framework for data fusion. Ecology.

[54] Dorazio R.M. (2014). Accounting for imperfect detection and survey bias in statistical analysis of presence-only data. Glob. Ecol. Biogeogr..

[55] Manica M., Caputo B., Screti A., Filipponi F., Rosà R., Solimini A., della Torre A., Blangiardo M. (2019). Applying the N-mixture model approach to estimate mosquito population absolute abundance from monitoring data. J. Appl. Ecol..

[56] Jarošík V., Kenis M., Honěk A., Skuhrovec J., Pyšek P. (2015). Invasive insects differ from non-invasive in their thermal requirements. PLoS ONE.

[57] Essl F., Lenzner B., Bacher S., Bailey S., Capinha C., Daehler C., Dullinger S., Genovesi P., Hui C., Hulme P.E. (2020). Drivers of future alien species impacts: An expert-based assessment. Glob. Chang. Biol..

[58] Huang D., Haack R.A., Zhang R. (2011). Does global warming increase establishment rates of invasive alien species? A centurial time series analysis. PLoS ONE.

