# Supplementary material for: Distribution Drivers of the Alien Butterfly Geranium Bronze (Cacyreus marshalli) in an Alpine Protected Area and Indications for an Effective Management
Source: Biology (Basel). 2022 Apr 7;11(4):563. doi: 10.3390/biology11040563 (PMC9027867; doi:10.3390/biology11040563)
Supplement: Supplementary file 1 [file biology-11-00563-s001.zip › biology-1639367-supplementary.pdf]

Supplementary Materials of Rocchia et al. 2022, *"Distribution drivers of the alien butterfly Geranium Bronze (Cacyreus marshalli) in an alpine protected area and indications for an effective management ".*

Table S1. Summary of the explanatory variables used for MaxEnt and N-mixture analysis.

**Variable name**	**Source**	**Resolution**	**Biological meaning**
Pelargonium abundance	Paradiso et al 2018	250x250 m	Species food plants may affect the abundances of the butterfly
Pelargonium in the neighbouring cells	Paradiso et al 2018	250x250 m	They may influence the concentration of eggs per plant
Annual Mean Temperature (BIO01)	Metz et al.2014	250x250 m	It may affect the butterfly life cycle and as a consequence the distribution
Temperature seasonality (BIO04)	Metz et al.2014	250x250m	It may affect the butterfly life cycle and as a consequence the distribution
Woodland cover	Local land cover map provided by the Park	Vector layer	The habitat structure could drive the butterfly site preferences and therefore its distribution
Ecotone cover	Local land cover map provided by the Park	Vector layer	The habitat structure could drive the butterfly site preferences and therefore its distribution
Grassland cover	Local land cover map provided by the Park	Vector layer	The habitat structure could drive the butterfly site preferences and therefore its distribution

Table S2. Details on the occurrence data used for Maxent analysis. The table reports the dataset sources (first column): National distribution dataset (CKmap) and Gran Paradiso CS project (CS GPNP); the accuracy; the number of occurrences in the study area; the period of investigations.

Dataset	Accuracy	N. occurrences	Year
CKmap	25 m	138	2015-2019
CS GPNP	25 m	3	2015-2019

Table S3. Model selection of the selected N-mixture model with different distributions for the λ parameter. K = number of parameters, AICc = Akaike's Information Criterion corrected for small sample, ΔAICc = AICc difference between the considered model and the best one, Wi = weight of each model, Cum.Wi = cumulative weight.

Models	mixtures	K	AICc	ΔAICc	W_i	Cum.Wi
ρ *pel_ava* λ *bio01 + pel_abu + pel_abu:bio01 + pel_neigh*	Negative Binomial	8	780.06	0	1	1
ρ *pel_ava* λ *bio01 + pel_abu + pel_abu:bio01 + pel_neigh*	Zero Inflated Poisson	8	877.49	97.43	< 0.01	1
ρ *pel_ava* λ *bio01 + pel_abu + pel_abu:bio01 + pel_neigh*	Poisson	7	979.54	199.48	<0.01	1

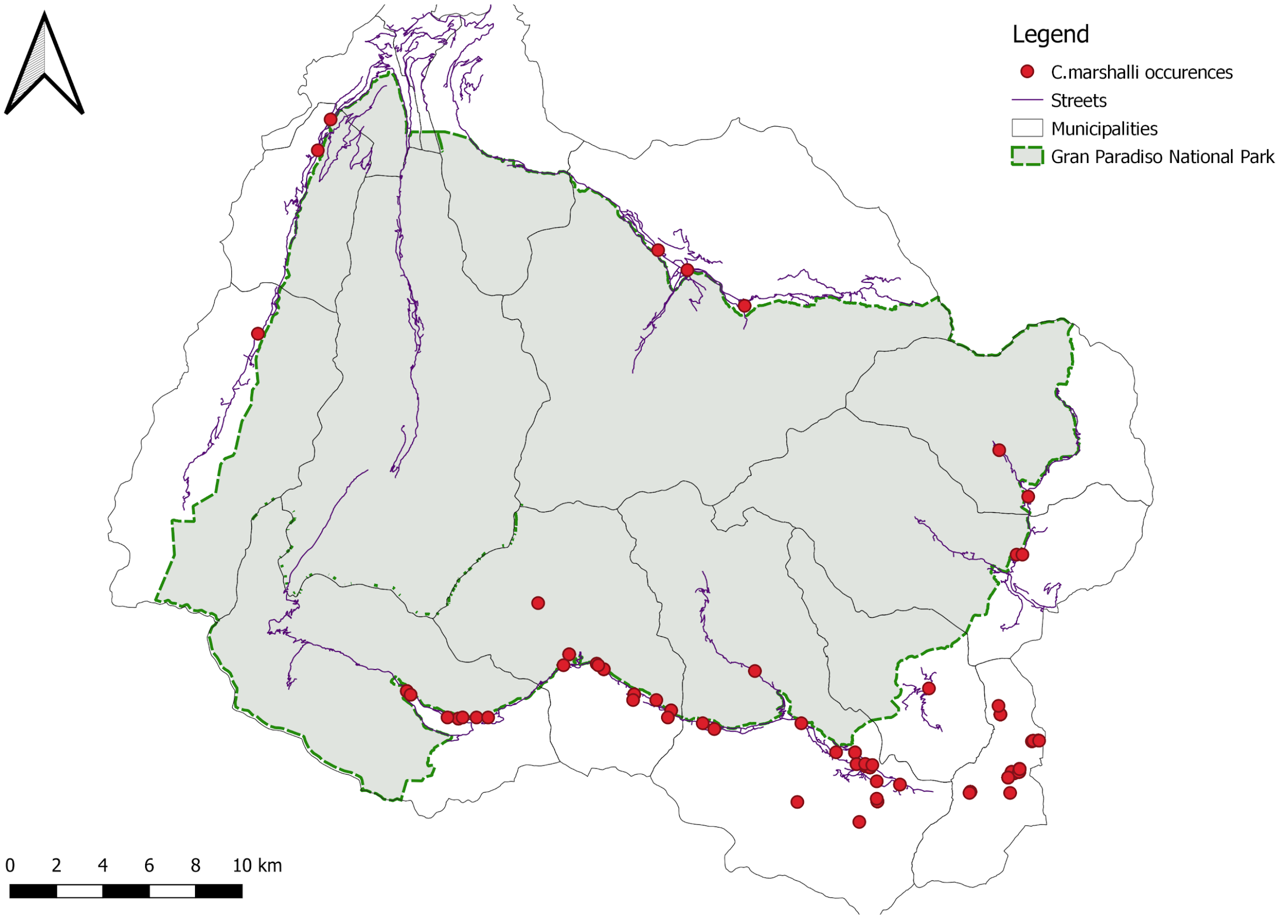

Figure S1. *Cacyreus Marshall* presence points. The map shows the opportunistic data of *C.marshalli* presence (red dots) in the study area.

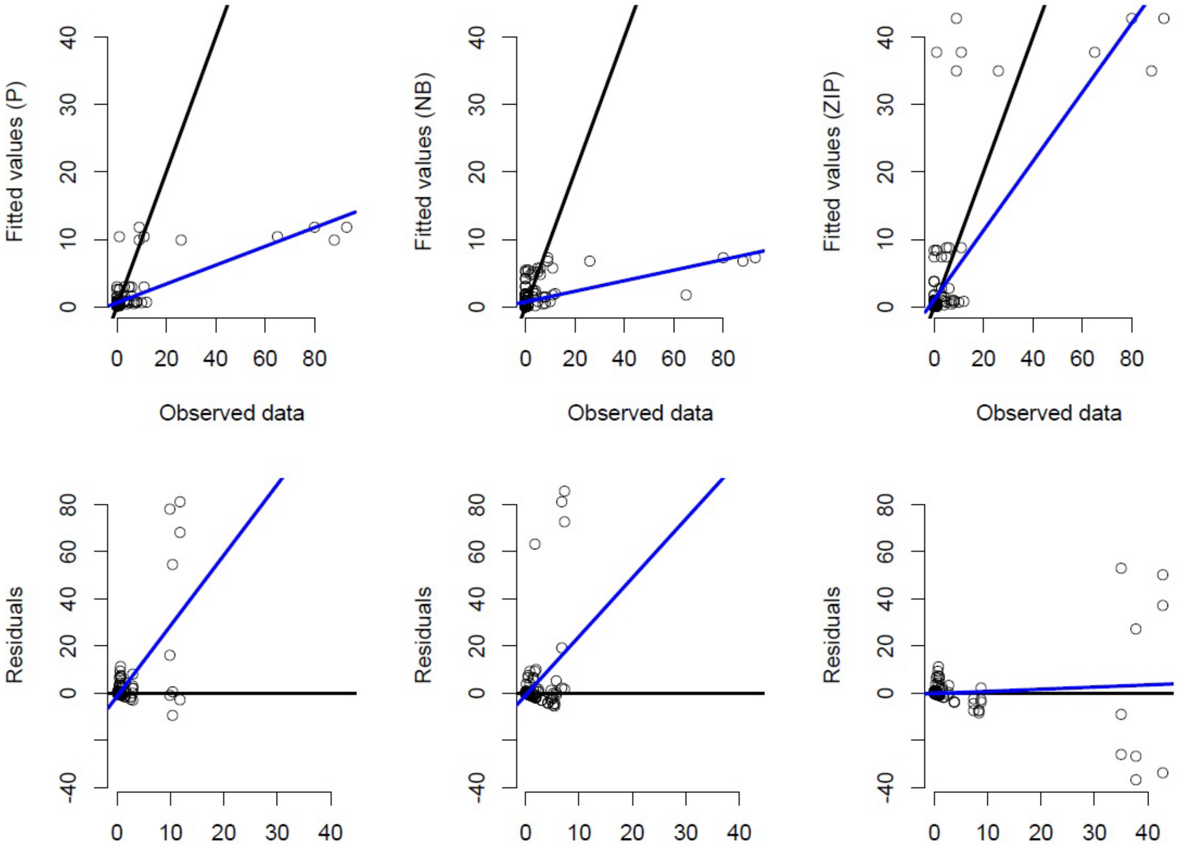

Figure S2. Model residual diagnostics for the N-mixture models with the three different distribution of λ (P = Poisson, NB = Negative Binomial, ZIP = Zero Inflated Poisson). a) the blue line represents the linear regression of the best fit and the black line a 1:1 relationship. b) black line shows the absence of residuals while blue line is the linear regression.
